# Fabrication and Characterization of PEEK/PEI Multilayer Composites

**DOI:** 10.3390/polym12122765

**Published:** 2020-11-24

**Authors:** Ángel Alvaredo-Atienza, Lu Chen, Verónica San-Miguel, Álvaro Ridruejo, Juan P. Fernández-Blázquez

**Affiliations:** 1 IMDEA Materials Institute, C/Eric Kandel 2, Getafe, 28906 Madrid, Spain; angelalvaredoatienza@gmail.com; 2 Fundación para la Investigación (FIDAMC), Desarrollo y Aplicación de Materiales Compuestos, Avda. Rita Levi Moltalcini 29, Getafe, 28906 Madrid, Spain; 3 Department of Materials Science, Universidad Politécnica de Madrid, ETSI Caminos. C/Profesor Aranguren, 3. 28040 Madrid, Spain; chenlualua@outlook.com (L.C.); alvaro.ridruejo@upm.es (Á.R.); 4 Department of Materials Science and Engineering and Chemical Engineering (IAAB), University of Carlos III of Madrid, Av. Universidad 30, Leganés, 28911 Madrid, Spain; vmiguel@ing.uc3m.es

**Keywords:** PEEK, PEI, multilayer, miscibility, glass transition, synchrotron radiation

## Abstract

Polyetheretherketone (PEEK)/polyetherimide (PEI) blends (50/50, *v/v*) keeping the crystal phase of PEEK have been manufactured by alternate PEEK/PEI layer stacking. This strategy avoided the complete miscibility of both polymers, keeping layers of PEEK and PEI unmixed along the sample thickness, as well as promoting the formation of a smooth interfacial layer where PEEK and PEI were mixed. The properties of this interface after processing at molten state and different times was studied by DSC, DMA, and X-Ray synchrotron. These techniques allowed monitoring the evolution of glass transition, where isolated T_g_’s for both pristine polymers were observed even after long processing time. PEEK crystallinity slightly decreased during manufacturing, whereas PEEK crystal parameters did not vary. These observations show that, although the interface—the zone where both polymers are mixed—grew, layers with pristine polymers remained even after prolonged processing time. The preservation of the PEEK crystallinity was also observed in the mechanical properties of the multilayer PEEK/PEI films, which were compared with pristine PEEK and PEI films. Multilayer samples processed for shorter times rendered higher young modulus, tensile strength, and strain at break.

## 1. Introduction

The development of technologically relevant polymer blends and polymer composites depends on the synergistic combination of two or more polymers with the aim to improve the final properties [1,2,3]. Sometimes, single polymer components do not reach the end use requirements, necessitating the use of a combination of two or more polymers. The properties of the final material depend strongly on the morphology, processing history, formulation, and interactions between the individual components [4]. One method to combine two or more polymers is the creation of a layered structure, which may lead to a broad range of improved properties. For over six decades, multilayer films have demonstrated their importance in the commercial marketplace due to their excellent optical, gas barrier, mechanical, or dielectric properties [3]. These unique properties have allowed multilayer films to be employed in several applications such as infrared (IR), visible, or ultraviolet (UV) light reflectors, films for toughness enhancing and selectivity filtering IR or UV radiation in glass window, flexible barriers in food packaging, medical applications, etc.

Polyetheretherketone (PEEK) is a high-performance engineering semi-crystalline thermoplastic polymer with remarkable mechanical properties and good chemical and temperature resistance [1], which provide a wide spectrum of commercial applications, including composites with carbon or glass fibers [4]. Polyetherimide (PEI) is a high-performance amorphous thermoplastic polymer with high strength and rigidity at high temperature, due to its high glass transition temperature of around 220 °C. However, the application of PEI has been limited due to its low solvent resistance [5].

Blends of PEEK/PEI have been studied due to their high miscibility, which can offer a chance to balance their properties, minimizing their respective drawbacks [1,2,5,6,7,8,9,10,11,12,13]. Applications in membranes and welding processes [14,15,16], as well as in the aerospace industry, where good mechanical properties at high temperatures are required [17], has increased the interest in PEEK/PEI blends. Several studies have demonstrated the high miscibility of both polymers in all blend compositions due to the identification of a single glass transition [2,5,10,12,17], which varied nearly as predicted by the Fox equation [5,17,18]. This high miscibility has an important influence in the crystallization of PEEK, because PEI polymer chains are rejected from PEEK crystalline regions, giving rise to two effects. First, the drastic decrease of crystallization rate of PEEK [5]. Later, the reduction of crystallinity in these PEEK/PEI blends, which is hindered completely, particularly at high cooling rates [12] when PEI content reaches 50% [5,12,17]. It is worth mentioning here the effect of crystallinity: it is well known that the best mechanical properties, thermal, and/or solvent resistance are not reached unless there is substantial crystallization [18,19,20,21]. Moreover, crystallinity plays an important role in the adhesion and mechanical properties of PEEK composites [22]. For this reason, preserving some degree of crystallinity is relevant for the potential applications of the blend, and a processing route based on multilayer stacking seems the most promising method to this end. As far as we know, this new strategy to keep high crystallinity in PEEK/PEI blends by processing them in multilayer films is reported here for the first time.

The aim of this study is to develop a new multilayer PEEK/PEI (50/50, *v/v*), which keeps the main characteristic properties of semi-crystalline PEEK, and adds the low stiffness and higher toughness of PEI, as well as its lower price. The multilayer films of PEEK/PEI were manufactured decreasing as much as possible the interdiffusion between PEEK and PEI during the processing. In addition, annealing process (at 380 °C for 120 min) was applied to PEEK/PEI multilayer in order to compare the properties after some degree of miscibility between both polymers. Furthermore, PEEK and PEI films were also manufactured to establish a comparison between the neat polymers and the multilayer material at different processing conditions to compare different grade of miscibility in the interface. DSC and synchrotron X-Ray measurements were carried out to study the thermal properties and semicrystalline morphology of multilayer films. Finally, thermo-mechanical and mechanical characterization were performed to study the final properties of the multilayer PEEK/PEI.

## 2. Materials, Processing, and Experimental Methods

### 2.1. Materials

Films 0.05 mm thick of PEEK (Victrex) and PEI (Ultem), manufactured by Goodfellow (Goodfellow Cambridge Ltd., Huntingdon, UK), were used in this work. PEEK is a semi-crystalline polymer, whose glass transition temperature, T_g_, is 145 °C and melting temperature, T_m_, is 340 °C. PEI is an amorphous polymer and its T_g_ is 215 °C.

### 2.2. Fabrication of PEEK/PEI Films

Multilayer PEEK/PEI films were manufactured by alternatively stacking two films of PEEK (50 μm) and two films of PEI (50 μm) and consolidating them with a hot plate press (LabPro 400, Fontijne Presses, Delft, The Nederlands). A 20 cm^2^ steel frame was used during manufacturing to control the thickness of the multilayer film. Variations in thickness were always lower than 10%. Two different kinds of films were manufactured at 380 °C for 10 min (PEEK/PEI@T380t10) and for 120 min (PEEK/PEI@T380t120). A force of 7 kN was applied in the last 5 min of each cycle and was kept during the cooling at 10 °C/min until room temperature was reached.

**Figure 1:**
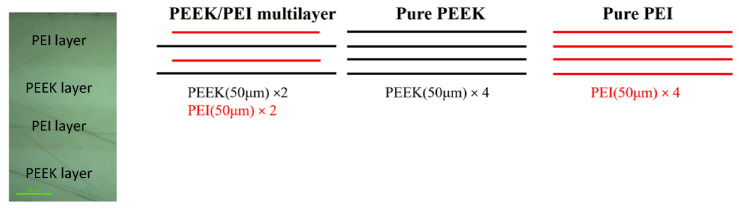
Stacking sequence of the manufactured films. Left: microscope image of the cross-section of a polyetheretherketone/polyetherimide (PEEK/PEI) multilayer specimen.

Films using the neat polymers (PEEK and PEI) were also manufactured following the same conditions and stacking 4 films for each polymer, as shown Figure 1.

### 2.3. Experimental Techniques

#### 2.3.1. Differential Scanning Calorimetry

The miscibility of PEEK/PEI samples and the thermal properties of PEEK, PEI, and PEEK/PEI samples were carried out with a DSC analyzer (TA Instruments, model Q200, New Castle, DE, USA).

PEEK/PEI multilayer samples were heated from RT to 400 °C at a rate equal to 10 °C/min and then held isothermally for 30 min. Afterwards, the sample was cooled from 400 to 20 °C at 10 °C/min. Using the same sample, the procedure previously explained was repeated six times to study the miscibility of PEEK/PEI in a multilayer sample.

PEEK and PEEK/PEI (@T380t10 and @T380t120) samples were processed by hot pressing. Then, DSC experiments were performed by heating to 380 °C at a rate of 10 °C/min and then held isothermally for 5 min. Later, the samples were cooled down from 380 °C to 20 °C at 10 °C/min.

The crystallinity (*X*_c_) of samples was calculated according to:

(1) $$X_{C}=\;\frac{\Delta H_{m}}{\left(1-\phi \right)\;\Delta H_{0}}$$

where Δ*H*_0_ is the heat of fusion of 100% crystalline PEEK, taken as 130 J/g [23], *ϕ* is the weight fraction per unit mass of nanoreinforcement and Δ*H_m_* is the melting enthalpy calculated by the integration of the melting endothermic peak for all samples.

#### 2.3.2. Synchrotron

Simultaneous wide-angle X-Ray diffraction (WAXS) and small-angle X-Ray scattering (SAXS) experiments were performed at the I22 beamline of the Diamond Light Source Synchrotron facility (Harwell Campus, Oxfordshire, UK). WAXS and SAXS data were collected on Pilatus P3-2M detectors, located for WAXS at a distance of 400 mm and at 4780 mm for SAXS. The beamline operated at a beam energy of 12.4 keV, giving a wavelength of 1 Å. Further details about this beamline are described in reference [24]. Data processing was done with the software DAWN (Diamond Light Source, Didcot, UK) [25].

SAXS correlation function was calculated with the following equation:

(2) $$\Gamma_{1}\left(z\right)=\frac{1}{Invariant}\int_{0}^{\infty }I\left(q\right)q^{2}\cos\left(qz\right)dq$$

Long spacing (L), average lamellar thickness (L_c_), and linear crystallinity (X_CL_) were calculated following the references [26], and by means of SasView software (https://www.sasview.org/).

#### 2.3.3. Dynamic Mechanical Analysis

The dynamic mechanical analysis (DMA) tests were performed on a DMA Q800 (TA Instruments, New Castle, DE, USA). Rectangle shape samples (10 × 3 × 0.2 mm^3^) were placed on tensile clamp. All tests were carried out at a heating rate of 2 °C/min from −140 °C to 250 °C, 5 Hz of frequency, and a strain of 20 μm.

#### 2.3.4. Mechanical Tests (Tensile Tests)

At least six samples were tested for each material in simple extension on an Instron 3384 testing machine (Instron, Norwood, MA, USA) at a constant crosshead speed of 10 mm/min. The dimension of the samples was 20 × 5 × 0.2 mm^3^. The elastic modulus was calculated from the linear region of the stress-strain curve (2% to 6% strain). Tensile strength and strain at break were also measured.

## 3. Results and Discussion

### 3.1. PEEK/PEI Interface Evolution with Different Processing Time at High Temperature

**Figure 2:**
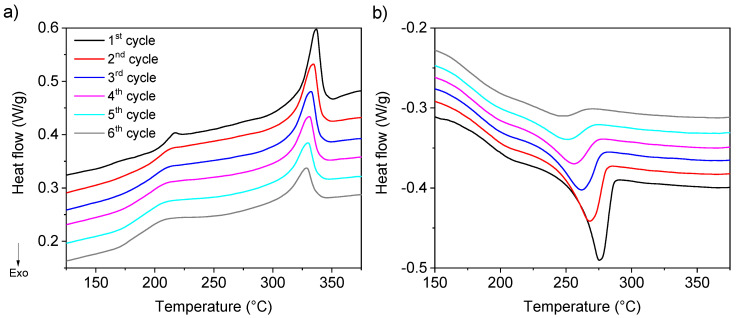
DSC thermographs of heating/isothermal at 400 °C for 30 min/cooling cycles, (**a**) heating (**b**) cooling.

**Figure 3:**
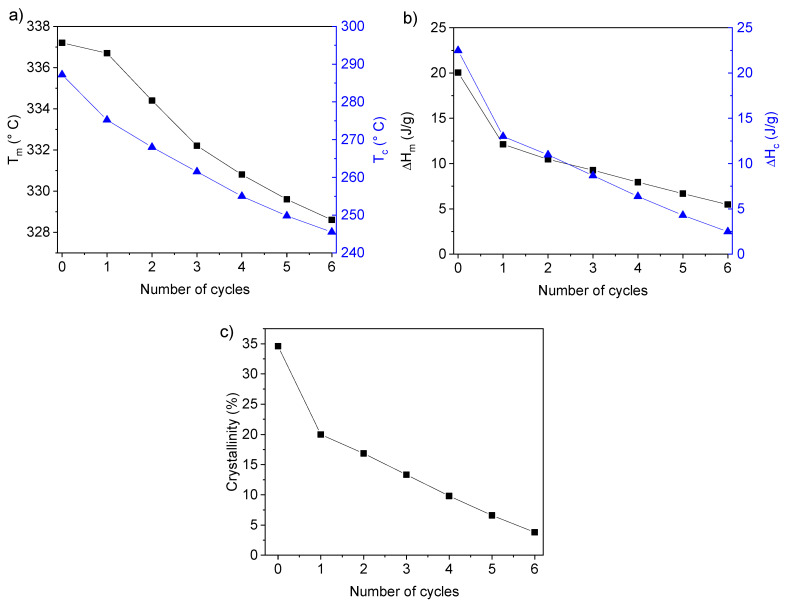
(**a**) Melting temperature (black squares) and crystallization temperature (blue triangles) *versus* number of heating/cooling cycles, (**b**) melting enthalpy and crystallization enthalpy against the number of processing cycle, and (**c**) degree of crystallinity against the number of heating/isothermal/cooling cycles.

The evolution of the interface in a PEEK/PEI multilayer after different annealing time at high temperature can be monitored by DSC. It is well known that the miscibility of the PEEK and PEI is high, the loss of crystallization capacity of PEEK being one of the consequences of this miscibility, as was mentioned above. In our multilayer material, high miscibility favored the interpenetration of chains between layers during the processing at high temperature. This fact ensures good adhesion between layers of PEEK and PEI due to the formation of an interface where PEEK and PEI are mixed. Therefore, in these multilayers of PEEK/PEI (50/50) the growing of the interface during the processing time at 400 °C, considering the interface as the region where PEEK and PEI coexist, has to be associated with a decrease of PEEK crystallinity, which can be followed by DSC. To study this phenomenon, a PEEK/PEI multilayer sample was manufactured at 380 °C for 10 min and was subjected to six cycles of heating/isothermal annealing/cooling from RT to 400 °C in the DSC. The duration of each isothermal annealing at 400 °C was 30 min. The heating and cooling DSC profiles of this experiment are shown in Figure 2. The endothermic peak associated to the melting of PEEK crystals appeared at lower temperature (Figure 3a) and with lower melting enthalpies (Δ*H_m_*) (Figure 3b) after each cycle. The same trend was found in crystallization temperature (T_c_) as well as in the crystallization enthalpies (Δ*H*_C_), but in this case the decrease in Tc was more intense, from 286 °C in the first cycle to 245 °C after the sixth cycle (Figure 2b). This behavior indicated that the crystallization kinetics was reduced when the annealing time at 400 °C was longer, as can be observed in the crystallinity grade of PEEK. During this time in molten state, the interpenetration of PEEK and PEI chains is favored, increasing the interface, considering this interface as the region where both PEEK and PEI chains coexist. The crystallization of PEEK in this interface is hindered by the presence of PEI. This effect has been previously reported on PEEK–PEI blends [11,27]. Harris et al. [2] observed that PEEK–PEI blends containing as little as 30% of PEEK were crystallizable but both crystallization kinetics and crystallinity itself were decreased [2,11]. For that reason, the global crystallinity of this PEEK/PEI multilayer was reduced almost by 75%, as proved by the fact that Δ*H_m_* diminished from 20 J/g, in cycle one, to around 5 J/g after six heating/cooling cycles (Figure 3b,c). The reduction in the crystallization kinetics was also evident, considering the decrease in the crystallization temperature observed with the number of the cycles (Figure 3a). This fact can produce changes in the crystal morphology, as will be discussed below. The formation of this interface region with a mixture of PEEK/PEI also affected the glass transition (T_g_). The two independent T_g_’s that would in principle be expected to appear during heating ramps were observed only after the first cycle at around 160 °C and 220 °C for PEEK and PEI, respectively. From the second cycle on, both T_g_’s tended to converge in just one at around 195 °C, reducing the temperature range in which the T_g_’s were observed along the cycle numbers. This behavior is common in miscible polymer blends.

A decrease in Δ*H_m_* and Δ*H*_c_ was observed increasing the annealing cycles due to the hindrance of PEEK crystallization posed by amorphous PEI (Figure 3b) because chain interpenetration occurred during the annealing time in molten state. Then, an interface with PEEK–PEI mixture was formed during this annealing at high temperature. This mixture in the interface caused the decrease of the enthalpy of melting (Δ*H_m_*) and the enthalpy of crystallization (Δ*H*_c_), as it has been observed when the PEI content of the PEEK/PEI blend reached 50% [18]. Crystallinity followed the same trend as Δ*H_m_* (Figure 3c).

### 3.2. Thermo-Mechanical Properties

**Figure 4:**
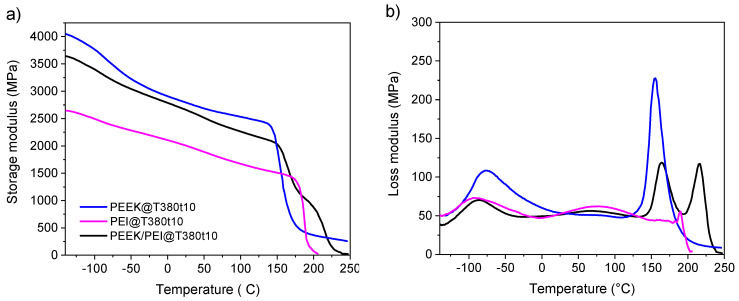
DMA plots of multilayer PEEK/PEI, PEEK and PEI (**a**) storage modulus and (**b**) loss modulus at 5 Hz.

Outside the interface, though, PEEK and PEI largely remain as neat polymers. The effect of keeping semicrystalline PEEK in a PEEK/PEI (50/50) blend thanks to this multilayer manufacture in thermo-mechanical properties was evaluated by Dynamic Mechanical Analysis (DMA). The storage modulus and loss modulus of PEEK, PEI and a PEEK–PEI multilayered compound are shown in Figure 4a,b, respectively. The storage modulus at 25 °C and T_g_ are summarized in Table 1 The pristine PEEK showed a storage modulus of 2797 MPa at 25 °C and a T_g_ of 149 °C. The storage modulus of PEI was much lower, 2000 MPa at 25 °C. However, the glass transition of PEI appeared at 179 °C. Our PEEK/PEI multilayer sample had a storage modulus close to the observed one in neat PEEK (2700 MPa at 25 °C) and, as expected for non-miscible polymer blends, two T_g_’s were observed at 154 and 206 °C. Therefore, this proposed PEEK/PEI multilayer morphology had two interesting effects: the first of them is that its storage modulus only decrease by a small extent. This is favored by the presence of semicrystalline PEEK. The second effect can be found at temperatures higher than 150 °C, where the PEEK/PEI multilayer showed greater storage modulus than neat PEEK and PEI until 220 °C, assisted by a non-expected increment in the glass transition for both components, more noticeable in the case of PEI. This phenomenon will be discussed later. This rise in mechanical performance at high temperature of the PEEK/PEI multilayer blend could have a high potential for future applications.

Figure 4b shows the profile of loss modulus for the three samples (PEI, PEEK, and PEEK/PEI multilayer), where secondary relaxations are observed. On the one hand, for PEI, the β process is centered at 77 °C attributed to the main chain oscillation involving the aromatic and benzimide rings [28]. The γ process was observed at −91 °C due to the oscillation of water and small segments of the polymer chains [28]. On the other hand, the γ process was observed at −77 °C in pristine PEEK, due to phenyl rings flips [29]. The β relaxation of the multilayer film was observed at 70 °C and was mainly caused by the PEI component. The γ relaxation of the multilayer was placed between the β relaxation of PEEK and the γ relaxation of PEI at −87 °C. Therefore, all the secondary relaxations of PEEK and PEI were observed in the multilayer films, but with small differences in the temperature as well as in the shape of the relaxations. We can infer that the relaxation behavior of both components was affected by the presence of each other. The main cause is probably the molecular interactions in the interface, due to the high miscibility of both components [28].

**Figure 5:**
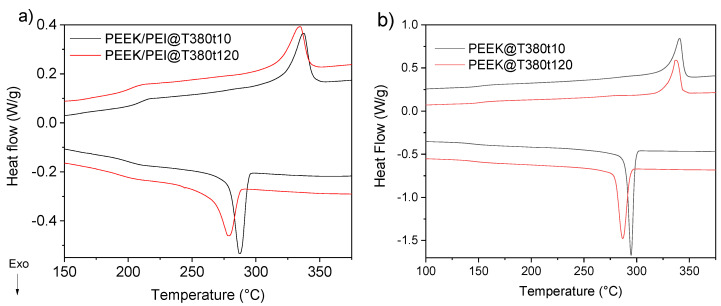
(**a**) DSC thermographs of PEEK/PEI multilayer and (**b**) DSC thermographs of PEEK films after 10 and 120 min at molten state (380 °C).

A second processing method was applied to the multilayer to favor the growth of the interface. The multilayer was kept at 380 °C for 120 min instead of 10 min. The DSC profiles of PEEK/PEI multilayer after 10 min and 120 min at 380 °C for processing cycle and cooled at 10 °C/min can be observed in Figure 5. Crystallization and melting temperature (T_c_, T_m_), together with degree of crystallinity (*X*_c_) of this multilayer are summarized in Table 2.

An increase in processing time produced a decrease in the melting temperature, as well as a wider and smaller endothermic peak, associated to the formation of smaller crystals. This effect is also observed in the cooling curve, where the differences in crystallization temperature and shape of the exothermic peak are more important. However, the decrease of the crystallinity is not so high, only around 5%, although the melting temperature and the shape of the melting endothermic peak, much broader with longer processing time, were quite different. This fact will be discussed in the next section, SAXS/WAXS characterization. The decrease of crystallinity was lower than the one observed in heating/cooling cycles for DSC analyses. This is due to the temperature of the annealing: in the case of DSC it was at 400 °C, whereas in the hot plate press was at 380 °C. Processing at lower temperature was introduced to minimize the thermal degradation of PEEK, which may irreversible change the physical and mechanical properties of PEEK [30]. Besides, this thermal degradation of PEEK occurs to a much larger extent in air than under nitrogen atmosphere [31]. Taking into account that the polymer in the hot plate press was exposed to normal atmosphere, whereas DSC was done under nitrogen atmosphere, it was decided to prepare the PEEK/PEI multilayer at 380 °C.

Though the crystallinity decrease is relatively low, the influence in the crystallization kinetics is relevant because the T_c_ decreased almost by 10 °C. This reduction in the crystallization rate was also observed in the PEEK/PEI after 10 min at 380 °C when compared to samples with only PEEK layers and the same processing method. Figure 5b shows the DSC profiles for the first heating and cooling for PEEK samples. Values of T_c_, T_m_, Δ*H*, and *X*_c_ for PEEK/PEI multilayer compound and PEEK alone are summarized in Table 2. The processing time had no influence on crystallinity and melting behavior. However, the crystallization temperature was reduced by around 8 °C. This hindered crystallization could be due to the beginning of thermal degradation [32]. Back to the PEEK/PEI multilayer, its crystallization temperature, as well as the onset of the exothermic crystallization peak in the sample processed during 10 min at 380 °C, was 8 °C lower than that for pure PEEK, without significant reduction of the crystallinity. Therefore, just from the initial formation of the PEEK/PEI interface, the kinetics of the crystallization is reduced, though the interface size should be small and then the crystallinity in the PEEK layer was not reduced. However, when the size of the interface increased, (case of samples processed at 380 °C for 120 min), crystallization kinetics and degree of crystallinity were reduced as well. This fact can be explained due to the increase of the PEEK/PEI interface, but also with a possible thermal degradation of PEEK, as it was commented above. FTIR microscopy and nanoindentation experiments in the cross-section of the PEEK/PEI multilayer will be performed in the near future to study the size and the chemical composition of the interface.

**Figure 6:**
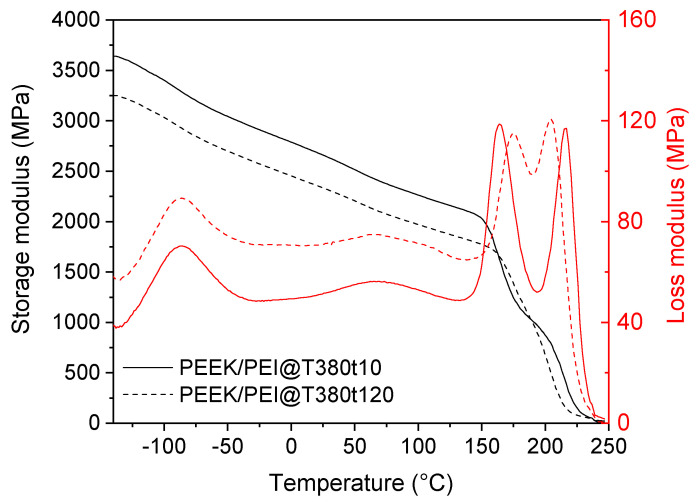
DMA plots of PEEK/PEI multilayer film after first and second cycle at 5 Hz. Storage modulus (black line), and loss modulus (red line).

Finally, the influence of the processing time at 380 °C on the thermo-mechanical properties was analyzed by DMA. Figure 6 shows the storage and loss modulus of PEEK/PEI multilayers after the annealing at 380 °C for 10 or 120 min. A decrease of storage modulus was observed for the longest processing time, due to the reduction of the crystallinity associated to the higher PEEK/PEI interface. Two T_g_’s could be observed in both samples (Figure 6) and two separate relaxation peaks were observed in loss modulus profile, indicating that the PEEK/PEI multilayer were not homogeneously blended. However, the difference between both T_g_’s decreased in the sample annealed at 380 °C for 120 min, indicating higher degree of mixture due to the growth of the interface, but layers of pure PEEK and PEI are still preserved in this multilayer film.

### 3.3. SAXS/WAXS Characterisation

**Figure 7:**
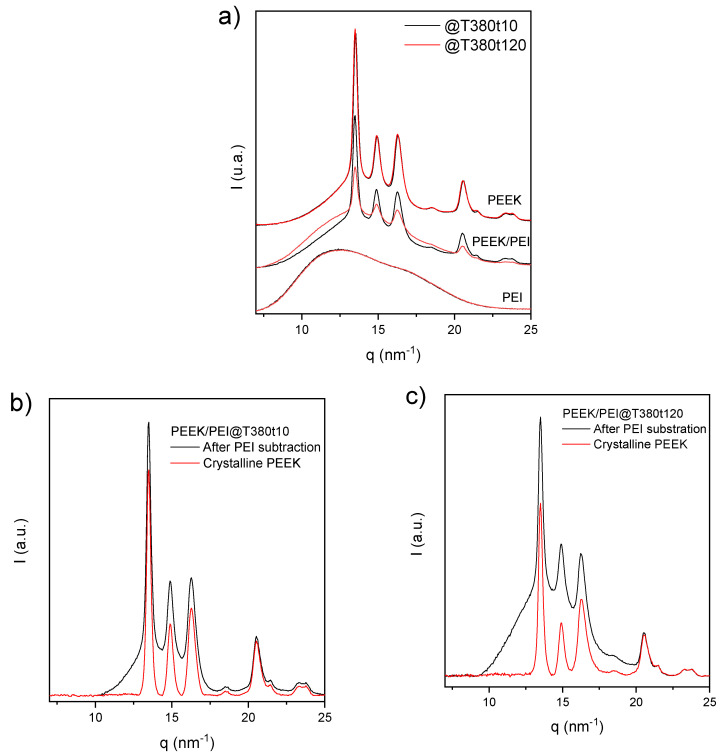
(**a**) Diffractograms of pristine PEEK and PEI and multilayer PEEK/PEI processed at 380 °C for 10 and 120 min. (**b**) Diffractograms of PEEK/PEI manufactured at 380 °C for 10 min after the subtraction of 50% normalized PEI diffractogram (black) and amorphous region of PEEK (red). (**c**) Diffractograms of PEEK/PEI manufactured at 380 °C for 120 min after the subtraction of 50% normalized PEI diffractogram (black); and remained crystalline PEEK (red) after subtraction of PEI and amorphous PEEK.

The effect of annealing time at a certain processing temperature was studied by SAXS/WAXS experiments. Figure 7 shows the WAXS X-Ray diffractograms for multilayer PEEK/PEI samples manufactured at 380 °C for different times. Diffractograms of pristine PEEK and PEI processed with the same processing cycles are provided for comparison. The results show that the annealing time did not affect the orthorhombic crystal phase of PEEK, whose three main characteristic diffraction peaks, (110), (111) and (200), were clearly observed at scattering vector (q) positions of 13.5, 14.9 and 16.3 nm^−1^, respectively, in both samples.

However, the effect in the PEEK/PEI multilayer is evident. The width and area of the amorphous halo increased after processing for 120 min, because the PEEK/PEI interface, mainly amorphous, must be added to the amorphous pure PEI layers. Therefore, the pure PEEK layer reduced its thickness and crystalline peaks were smaller, providing a lower global crystalline fraction. In order to analyze the crystalline morphology in the isolated PEEK layer, the amorphous halo of PEI was subtracted; taking into account that the multilayer is composed by layers of 50 μm of PEEK and PEI, the amorphous halo subtracted was normalized to the half of the diffractogram of PEI (Figure 7a). These diffractograms for PEEK regions are shown in Figure 7b for PEEK/PEI multilayer processed for 10 min and in Figure 7c for a sample processed for 120 min. Finally, to obtain the pure crystalline phase of PEEK, the broad amorphous halos were simulated and subtracted from previous diffractograms. The ratio between the areas of the profiles in Figure 7b,c can be considered the crystallinity in the PEEK layer, whose values were 45% and 30% for multilayers processed during 10 and 120 min, respectively. This important decrease of crystallinity may be justified by the reduction of the thickness in the pure PEEK layer, due to the growth of the interface PEEK/PEI, which is amorphous. Its progress was clearly observed by the wider amorphous halo observed with the processing time, such as in original multilayer diffractograms as in the profiles after subtraction of 50% normalized PEI diffractograms. Finally, if extracted crystalline diffractograms of both multilayers are compared to each other, no important differences were observed for the crystalline phase of PEEK. As it was mentioned above, both samples presented the three main diffraction of orthorhombic crystal phase of PEEK. However, there were small differences in the width of the diffraction peaks, being particularly in the (200) peak, at q = 16.3 nm^−1^, where this difference is more important. This width is related to the correlation length obtained by Scherrer equation (K∙2π/w_(200)_, being w_(200)_ the width at half-height of the 200 reflection peak in q (scattering vector), and considering a value of 1 for K (shape factor)). After applying this equation, a correlation length of 11.7 nm for PEEK/PEI@T380t10 and 9.81 nm for PEEK/PEI@T380t120 were obtained, respectively. This implies that the size of the PEEK crystal was slightly reduced under the longer processing time. This fact can be evaluated by SAXS as well.

**Figure 8:**
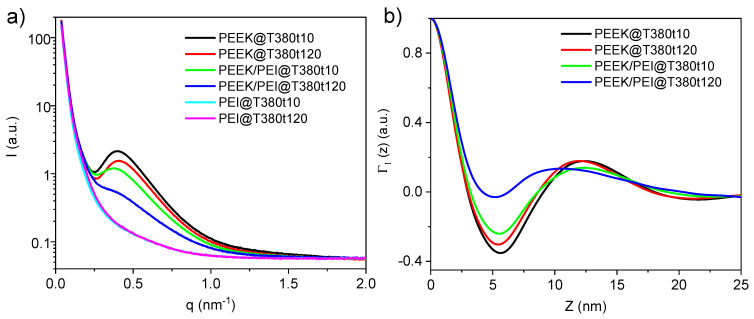
(**a**) SAXS patterns of all samples. (**b**) The correlation functions Γ_1_(z) for scattering data obtained for PEEK and PEEK/PEI samples at different processing times.

The original SAXS patterns of all samples are shown in Figure 8a. SAXS peaks appeared only in the samples which contained PEEK. The intensity of these peaks was higher for the samples of pure PEEK, and the increase of processing time reduced the intensity of the peak and shifted its position towards higher q. The correlation functions were calculated from the data displayed in Figure 8b*,* Equation (2) was used to calculate the lamellar variables: L (long spacing), L_c_ (average lamellar thickness), and X_CL_ (linear crystallinity). These data are summarized in Table 3.

The processing time for pure PEEK has a barely noticeable influence, since only a really small decrease in L and L_c_ was observed, without variation of linear crystallinity. Thus, the thermal degradation that occurs during the annealing at high temperature, which is reported to affect the rheological behavior of the polymer [33], has not effect in the crystalline morphology. In the case of PEEK/PEI, the sample with 10 min of processing time exhibited similar L, L_c_, and *X*_CL_ to neat PEEK, demonstrating that the mixture of PEEK and PEI is just limited to the interface. Therefore, a certain thickness of pure PEEK remained in these samples. After 120 min, both the long spacing and lamellar thickness were slightly reduced, but the linear crystallinity had similar values to PEEK. This fact, and taking into account that global crystallinity decreased as observed by DSC and WAXS, proves the existence of a region where PEEK remained essentially pristine or with a very low influence of PEI. FTIR and nanoindentation experiments will be performed in near future to analyze in greater details the distribution of PEEK and PEI in the multilayer composites.

### 3.4. Mechanical Properties

**Figure 9:**
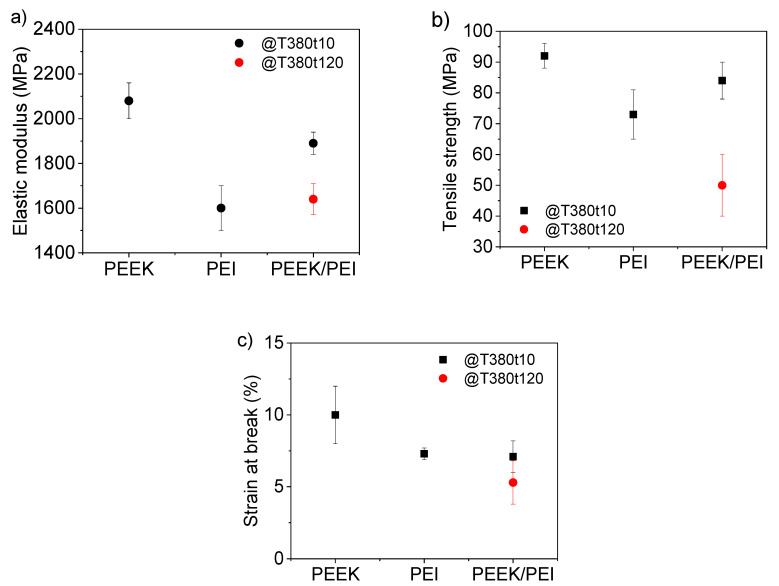
(**a**) Elastic modulus, (**b**) tensile strength, and (**c**) strain at break of PEEK, PEI, and PEEK/PEI samples after processing for 10 and 120 min.

Tensile tests were carried out on PEEK, PEI, and PEEK/PEI specimens manufactured with different processing times. Elastic modulus, tensile strength, and strain at break of the samples are displayed in Figure 9. The mechanical properties of PEEK, as expected, showed the highest performance compared to PEEK/PEI and PEI samples. Nevertheless, the PEEK/PEI blend consolidated just 10 min at 380 °C also exhibited remarkable mechanical properties, with a minimal decrease equal to 6% and 9% in the elastic modulus and tensile strength, respectively, with respect to neat PEEK. Thus, the mechanical properties of the multilayer were significantly closer to PEEK properties than to those of PEI. This behavior is different to previously reported results in PEEK–PEI blends, where the mechanical properties showed a clear degradation with respect to those of PEEK due to the loss of crystallinity [34,35]. Therefore, the PEEK/PEI multilayer structure presents a clear advantage regarding mechanical properties compared to standard blends where polymers are fully mixed.

In addition to this, PEI and PEEK/PEI samples showed the same strain at break (7.1%), which suppose a decrease of 30% compared to neat PEEK. The multilayer structure of PEEK/PEI material with two separate phases inherited properties from both of them: cracks nucleate in the less ductile polymer and once the PEI layer failed, the multilayer PEEK/PEI could not carry the total load and broke. Nevertheless, the obtained value for strain at break is suitable for most engineering applications.

Rather predictably, the PEEK/PEI multilayer processed at 380 °C for 120 min displayed a certain drop in its mechanical properties, such as the elastic modulus and tensile strength. This fact could be explained due to the higher degree of mixture between PEEK and PEI, higher interface thickness (mixed PEEK–PEI region), and lower global crystallinity.

## 4. Conclusions

Homogeneous blends of PEEK, which is a semicrystalline polymer, with PEI—an amorphous one—results in a mixture with moderate mechanical properties, not suitable for demanding structural applications. The main reason for this drop in the mechanical properties is the loss of crystallinity in PEEK due to the interpenetration of the molecular chains of both polymers, which hinders a compact packing of PEEK chains. In order to take advantage of the miscibility of both polymers while preserving the crystallinity of PEEK, alternate PEEK/PEI multilayer stackings were manufactured with a hot plate press. On the one hand, the good miscibility of PEEK and PEI favored the interpenetration of chains between layers over a certain thickness during the processing at molten state, creating an interfacial region where both polymers coexist. This interface ensured the good adhesion among layers. It has been observed that longer processing time reduced the PEEK crystallinity, crystallization temperature, and the evolution from two separated glass transitions to a single one with broader temperature range, due to the coexistence of regions with isolated polymers and interface whose glass transition was in between T_g_’s for pure polymers. This evolution of the glass transition was clearly observed in the DMA profile as well. Therefore, it can be concluded that the processing time determined the size of this interface. Outside this region, both PEEK and PEI are supposed to be preserved as pristine polymers.

To validate this hypothesis (i.e., that the crystalline morphology of the PEEK was not altered by the processing time and/or the presence of PEI layer), the multilayer was studied by means of WAXS and SAXS. Long spacings, lamellar thicknesses, linear crystallinities, and crystallization temperatures showed not very different values in pristine PEEK samples as well as PEEK/PEI multilayer materials. Therefore, unaltered PEEK regions can be found in the multilayer PEEK/PEI samples. The global reduction of crystallinity is due to the reduction of the thickness (and consequently, the volume) of these pristine PEEK regions.

PEEK/PEI multilayer composites processed for a short time (10 min) at 380 °C showed a storage modulus closer to PEEK than to PEI. Moreover, the storage modulus at temperature over 150 °C of the PEEK/PEI multilayer material was higher than pure PEEK, probably due to the remaining crystal phase of PEEK combined with high glass transition of PEI. This fact did not happen in homogeneous PEEK–PEI blends, and it could be relevant for potential applications. The mechanical properties were affected by the PEEK crystallinity in the PEEK/PEI multilayer composites. As a result, elastic modulus, tensile strength, strain at break, and storage modulus measured by DMA showed a certain degradation when they were manufactured with longer (120 min) permanence at 380 °C, because crystallinity was lower in this case. However, multilayer specimens processed with shorter (10 min) permanence time at the same temperature displayed excellent mechanical properties, notably close to those of PEEK in terms of elastic modulus and strength. These properties, together with a reasonable value for fracture strain (≈7.1%), and the competitive price of PEI make multilayer PEEK/PEI composites an extremely promising polymer for structural applications.

## Figures and Tables

**Table 1:** DMA results at 5 Hz.

Material	Storage Modulus (25 °C)	T_g_ (Onset)	T_g_ (Peak)	β Peak (°C)	γ Peak (°C)
PEEK	2797	149	155	−76.5	
PEI	2006	179	-	77.4	−91.2
PEEK/PEI	2657	154/206	165/216	69.9	−86.7

**Table 2:** DSC results of PEEK and PEEK/PEI multilayer films processed at 380 °C for 10 or 120 min.

Material	T_c_	T_m_	Δ*H_m_*	*X*_c_ (%)
PEEK/PEI@T380t10	287.2	337.6	22.6	34.6
PEEK/PEI@T380t120	278.6	335.4	19.4	29.8
PEEK@T380t10	295.1	340.8	45.1	34.7
PEEK@T380t120	287.1	337.2	44.9	34.5

**Table 3:** Long spacings, lamellar thicknesses, and linear crystallinities obtained from correlation functions of PEEK and PEEK/PEI samples.

Sample	Processing Time at 380 °C (min)	Long Spacing (L, nm)	Lamellar Thickness (L_c_, nm)	Linear Crystallinity (*X*_CL_)
PEEK	10	12.4	3.64	29.3
PEEK	120	12.0	3.51	29.3
PEEK/PEI	10	12.4	3.54	28.7
PEEK/PEI	120	10.6	3.31	31.4
